# Right atrium thrombosis in nonvalvular permanent atrial fibrillation

**Published:** 2011-11-24

**Authors:** A. Bălăceanu

**Affiliations:** “Carol Davila” University of Medicine and Pharmacy, Bucharest Ilfov Clinical Hospital, Internal Medicine Department, Bucharest Romania

**Keywords:** cardiac arrhythmia, echocardiography

## Abstract

Nonvalvular atrial fibrillation is the most common sustained arrhythmia in adults, and it is described as a relationship between it and right atrium thrombosis. A case of a 76–year–old man who presented with severe recent-onset dyspnea and several co–morbidities, such as permanent atrial fibrillation, with no anticoagulant therapy is reported here. Echocardiography showed a massive thrombus in right atrium, without any clinical or echographic signs of peripheral veins thrombosis. This article is also a review of the cases from literature.

## Background

Nonvalvular atrial fibrillation(AF) is the most common sustained arrhythmia in adults and the lack of an effective atrial contraction results in blood stasis and the formation of potentially embolic atrial thrombi. Many authors observed thrombi in the both atria to the patients with nonvalvular permanent AF, with a high prevalence in the left atrium and the left appendage.

## Case presentation

A 76–year–old man presented to the emergency department with severe recent–onset dyspnea. He had a medical history of high blood pressure, stroke, myocardial infarction, permanent atrial fibrillation, NYHA class II left ventricular failure. The patient had no anticoagulant therapy, only antithrombotic treatment. Clinical exam revealed decreased vesicular murmur, especially in left side, severe dyspnea, irregular rhythm, without signs of peripheral deep veins thrombosis. Blood tests were in normal limits, except hypercholesterolemia (278mg/dl). ECG: atrial fibrillation, QS in inferior leads. Two–dimensional transthoracic echocardiography showed a massive thrombus, highly mobile, from the right atrium prolapsing to the right ventricle toward the tricuspid valve in diastole (Figure A, B), back motion of the thrombus in right atrium in the systole, right ventricular free wall hypokinesis, and severe tricuspid regurgitation.

Depressed left ventricular function with an ejection fraction of ≈45% was observed. Chest x–ray showed opacity in the lower half of left lung. Abdominal, pelvic, peripheral veins ultrasound was in normal limits. The patient went quickly into hemodynamic collapse despite the intensive therapy. The family refused the necropsy.

**Figure A, B:**
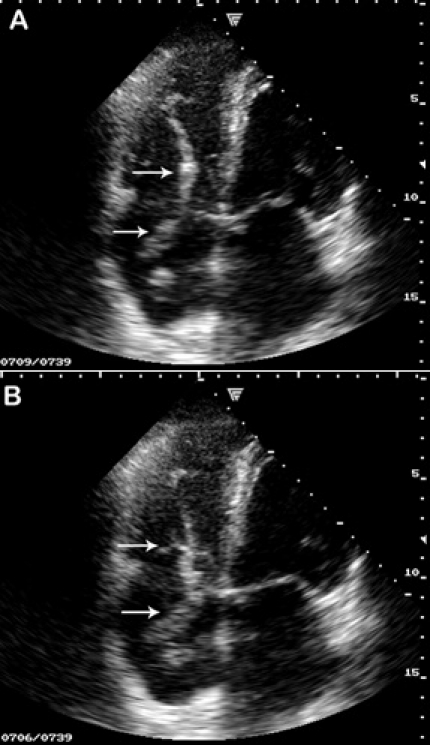
Two-dimensional transthoracic echocardiography

## Discussions

The guidelines on diagnosis and management of acute pulmonary embolism recommend anticoagulation therapy to the patients with predisposing risk factors like deep venous thrombosis, active cancer, prolonged bed rest, obesity, and thrombophilia. But our patient did not have these risk factors. He had permanent atrial fibrillation, with indication of anticoagulant treatment, but he received antithrombotic treatment (aspirin 75 mg/day), beta blocker, and converting enzyme inhibitor.

More recently findings in clinical and experimental studies have suggested a possible role of inflammation in the pathogenesis of chronic atrial fibrillation and its thrombotic complications [**1**], and diffuse biatrial disease, with a complex histopathological substrate characterized by HCN4–/PAS–positive cardiomyocites, ICLC scattered in fibrotic tissue, inflammatory infiltrate and sympathetic nerve structures in the atria and the pulmonary vein muscle sheeves.[**2**] A considerable number of patients required ablation in the right atrium and the cava superior to achive AF termination, indicating a biatrial substrate of chronic AF. Despite the fact that the ablation procedures with the intention to cure AF predominanly concentrated on the left atrium and pulmonary veins, it was demonstrated by biatrial multielectrode mapping that the activation gradient from the left atrium to the right atrium is characteristically for paroxysmal AF, but not for chronic AF.[**3**]

The subject is still controversial, some authors considering that AF is associated with an increased risk of thrombus formation in the left but not in the right atrium, because left atrial platelet reactivity is increased compared to the right atria and peripheral circulation.[**4**]

In a large study Pesavento assessed the prevalence of heart disease in 11236 consecutive patients older than 60 years, discarged from the hospitals with a diagnosis of pulmonary embolism and observed a higher prevalence of all–cause heart diseases in patients with a diagnosis of pulmonary embolism nonassociated with deep vein thrombosis, generating the hypotesis that some specific heart disease in older patients could themselves be a possible source of pulmonary emboli.[**5**]

The most frequently encountered symptom in pulmonary embolism is moderate–severe dyspnea. However many patients are smokers, elderly and have multiple co–morbidities, especially COPD, arterial hypertension, cardiac failure, dyslipidemia, diabetes, coronary and peripheral arterial disease with some degree of dyspnea. If dyspnea is severe, the patient seek medical advice, but if it is more or less chronic, specialist’s advise may be delayed. Anyway echocardiographic assessment is indicated for any patient presenting with unexplained, sudden severe dyspnea, cardiovascular collapse or syncope.

There are a lot of cases described in the literature, especially as echocardiographic data. De Divitiis showed that patients with nonvalvular AF had lower tricuspid annular excursion, larger right atrium chamber area, larger right appendage and right atrium ejection fraction and peak emptying velocities lower than in those with sinusal rhythm, with atrial thrombi in both sides.[**6**]

Subramanian considered that AF is associated with depressed right and left atrium appendage ejection velocities. The larger right atrium appendage width and lack of anatomic remodeling may partially explain the substantially lower prevalence of right atrium appendage thrombus found among patients with AF.[**7**]

Sahin demonstrated that in patients with permanent AF, impairement of right atrium apendage function and developement of right atrial spontaneous echo contrast-thrombus are closely related to the underlying etiology, and it was observed in 75% of the mitral stenosis patients, in 25% of hypertensive patients and in 30% of hyperthyroidism patients.[**8**] Although relatively rare when compared to the left side, right atrium appendage thrombus has also the potential of embolism and should be screened. Ozer described a case of right atrium appendage thrombus in wich the left atrium and left atrium appendage were spared [**9**] and Sonoda described multiple organised thrombi in the right atrium, interatrial septum and left appendage, to an old patient with history of hypertension and nonvalvular atrial fibrillation.[**10**] Kitayama identified thrombi in left atrium, right atrium and in both atria in the patients with AF, scaned by ultrafast CT, but only in half of these patients only in left atrium, when the patients were evaluated by echocadiography.[**11**] Collins founded atrial thrombi in the both atria and both appendages to the transesophageal echocardiography, more frequently in the left side.[**12**]

Many authors showed that there are two types of right atrium thrombi: type A, worm or serpentine shape or, extremely mobile, originated in deep veins of the pelvis or legs, and type B, small, parietal and immobile. Fixed right atrial thrombi may well arise in situ, although the localizing stimulus for their formation is not obvious and the incidence and distribution of the underlying disease status appear similar to those of patients with mobile thrombi.[**13**,**14**,**15**]

AF could affect both atria in nonvalvular AF, in contrast with valvular AF. Bilge founded right atrium appendage thrombi in 7.5% of the patients with chronic AF treated with warfarine.[**16**] In other studies right atrial spontaneous echo contrast has a prevalence of 14% in patients with atrial arrhythmia who undergo transesophageal echocardiography (TEE)–guided conversion,[**17**] and the incidence of perfusion defects in pulmonary scintigraphy was significantly higher in the group with spontaneous contrast in right atrium than in the group without it.[**18**] Others described right atrial thrombi in fewer than 1% of patients with atrial arrhythmia.[**17**]

In a population-based study of 23796 consecutive autopsies Oqren showed that right atrial thrombosis is as common as left cardiac thrombosis.The diagnosis should be considered in all cases of pulmonary embolism, especially in patients with atrial fibrillation or myocardial infarction and the absence of confirmed deep vein thrombosis.[**19**]

In our case, there was a massive thrombus, highly mobile, in the right atrium, without clinical or echographic signs of deep vein thrombosis to an elderly patient with multiple co–morbidities.

## Conclusions

The relation between atrial fibrillation, cardiac failure, arterial hypertension and pulmonary embolism need care about therapy, especially to the elderly people. A relatively high incidence of pulmonary embolism among patients with chronic atrial fibrillation not treated with anticoagulants or with poorly controlled anticoagulation therapy was noted by different authors. I consider that we need to establish the causal relationship between right atrial thrombus and permanent nonvalvular atrial fibrillation, despite the fact that the risk of systemic embolism in atrial fibrillation is, in itself, sufficient reason to recommend anticoagulant therapy.
